# Geographic Variation in Appointment Wait Times for US Military Veterans

**DOI:** 10.1001/jamanetworkopen.2022.28783

**Published:** 2022-08-25

**Authors:** Yevgeniy Feyman, Daniel A. Asfaw, Kevin N. Griffith

**Affiliations:** 1 Department of Health Law, Policy and Management, Boston University School of Public Health, Boston, Massachusetts; 2 Partnered Evidence-Based Resource Center, VA Boston Healthcare System, Jamaica Plain, Massachusetts; 3 Department of Health Policy, Vanderbilt University Medical Center, Nashville, Tennessee

## Abstract

**Importance:**

Timely access to medical care is an important determinant of health and well-being. The US Congress passed the Veterans Access, Choice, and Accountability Act in 2014 and the VA MISSION (Maintaining Systems and Strengthening Integrated Outside Networks) Act in 2018, both of which allow veterans to access care from community-based clinicians, but geographic variation in appointment wait times after the passage of these acts have not been studied.

**Objective:**

To describe geographic variation in wait times experienced by veterans for primary care, mental health, and other specialties.

**Design, Setting, and Participants:**

This is a cross-sectional study using data from the Veterans Health Administration (VHA) Corporate Data Warehouse. Participants include veterans who sought medical care from January 1, 2018, to June 30, 2021. Data analysis was performed from February to June 2022.

**Exposures:**

Referral to either VHA or community-based clinicians.

**Main Outcomes and Measures:**

Total appointment wait times (in days) for 3 care categories: primary care, mental health, and all other specialties. VHA medical centers are organized into regions called Veterans Integrated Services Networks (VISNs); wait times were aggregated to the VISN level.

**Results:**

The final sample included 22 632 918 million appointments for 4 846 892 unique veterans (77.3% male; mean [SD] age, 61.6 [15.5] years). Among non-VHA appointments, mean (SD) VISN-level appointment wait times were 38.9 (8.2) days for primary care, 43.9 (9.0) days for mental health, and 41.9 (5.9) days for all other specialties. Among VHA appointments, mean (SD) VISN-level appointment wait times were 29.0 (5.5) days for primary care, 33.6 (4.6) days for mental health, and 35.4 (2.7) days for all other specialties. There was substantial geographic variation in appointment wait times. Among non-VHA appointments, VISN-level appointment wait times ranged from 25.4 to 52.4 days for primary care, from 29.3 to 65.7 days for mental health, and from 34.7 to 54.8 days for all other specialties. Among VHA appointments, wait times ranged from 22.4 to 43.4 days for primary care, from 24.7 to 42.0 days for mental health, and from 30.3 to 41.9 days for all other specialties. There was a correlation between wait times across care categories and setting (VHA vs community care).

**Conclusions and Relevance:**

This cross-sectional study found substantial variation in wait times across care type and geography, and VHA wait times in a majority of VISNs were lower than those for community-based clinicians, even after controlling for differences in specialty mix. These findings suggest that liberalized access to community care under the Veterans Access, Choice, and Accountability Act and the VA MISSION Act may not result in lower wait times within these regions.

## Key Points

**Question:**

How long do veterans wait when seeking various types of medical care throughout the US?

**Findings:**

In this cross-sectional study of more than 22 million appointments for nearly 5 million veterans, substantial geographic variation in appointment wait times was observed across 3 care categories: primary care, mental health, and all other specialties. Veterans Health Administration wait times were lower than those for community-based clinicians in many areas even after controlling for differences in specialty mix.

**Meaning:**

These findings suggest that policies intended to increase veterans’ access to community-based clinicians may be insufficient to lower wait times in many areas of the country.

## Teaser

This cross-sectional study describes geographic variation in wait times experienced by veterans for primary care, mental health, and other specialties.

## Introduction

Timely access to medical care is widely acknowledged as an important determinant of human health and well-being.^1^ However, the US continues to lag behind other wealthy nations in terms of both access and equity.^2^ The Veterans Health Administration (VHA) provides care to millions of veterans through a network of 171 medical centers and affiliated outpatient clinics.^3^ To improve access to care for veterans, the US Congress passed the Veterans Access, Choice, and Accountability Act in 2014 and the VA MISSION (Maintaining Systems and Strengthening Integrated Outside Networks) Act in 2018, both of which allow enrolled veterans to seek primary, emergency, mental health, and other specialty care in their communities, at VHA expense, if they meet certain access criteria.^2,4^ As part of MISSION Act criteria, if veterans are unable to receive primary care or mental health care within 20 days, or other specialty care within 28 days, they can qualify for receiving care in the community.^4^

The VHA now relies on community-based medical professionals to fill millions of appointment requests for hundreds of thousands of veterans each year. VHA has established contracts with Optum Public Sector Solutions and TriWest Healthcare Alliance to provide care to veterans using their existing networks,^5^ which include nearly 1 million medical clinicians.^6^ These appointments are typically reimbursed at Medicare rates,^7^ and VHA enforces network adequacy standards to ensure sufficient geographic coverage for both primary and specialty care within their network of community care clinicians.^6^

However, there is limited evidence to date on the geographic variation in veterans’ wait times for community care, or how these wait times compare to those for VHA medical centers. Prior research^8,9,10^ has described appointment wait times experienced by the general population for particular specialties or specific locations of the country, but these efforts often rely on labor-intensive survey efforts because of a lack of data availability and also rely on small, likely unrepresentative, sample sizes.

In this analysis, our objectives were to study geographic variation in the wait times that veterans experience when seeking care from both VHA and community-based clinicians. Wait times were divided into 3 care categories: primary care, mental health, and all other specialties. We also identified areas of the country where VHA wait times outperform the private sector, and vice versa. To our knowledge, this study is the first national comparison of appointment wait times experienced by US military veterans for both VHA and non-VHA clinicians. A priori, we hypothesized that the VHA would have lower wait times than community-based clinicians. Additionally, we provide our data as a resource for researchers who would like to examine wait times for specific specialties or geographic regions.

## Methods

This cross-sectional study was considered exempt by the VA Boston Healthcare System’s institutional review board and adheres to the Strengthening the Reporting of Observational Studies in Epidemiology (STROBE) reporting guideline for cross-sectional studies. Institutional policy provides a waiver of informed consent because the research, which includes millions of veterans, could not be practicably carried out otherwise.

### Data Source and Population

We leveraged recently released data from the VHA’s Corporate Data Warehouse to identify new consultation requests across a range of medical specialties.^11^ These referrals represent new clinical relationships, vs repeat visits for established patients. The VHA uses stop codes, which are 3-digit identifiers used to identify the work group primarily responsible for providing care and are used for purposes of managerial accounting and evaluation, to indicate specialty type. Note that throughout the text, we used the terms stop code and specialty interchangeably. Self-reported information on race and ethnicity was extracted from the data set for general demographic information, but race and ethnicity data were not used in any analyses.

Using a previously validated algorithm, we identified care referrals occurring between January 1, 2018, and June 30, 2021.^11,12^ We extracted information on appointment specialty, patients’ county of residence, when the referral was initially made, and when the appointment was completed. All referrals must first be approved by the local VHA medical center; wait times were thus calculated as the difference between the date of approval and the appointment date. By excluding this administrative burden, we facilitated a more apples-to-apples comparison between VHA and community care wait times. Consultations with missing data were excluded (<0.01% of total consultations). Our sample was further limited to stop codes with at least 1000 total consultations in both the VA and the community during 2018 to 2021.

VHA medical centers are organized into regions called Veterans Integrated Services Networks (VISNs). VISNs are independent VHA networks that operate under directors who have autonomy to manage staffing and administrative capacity in a decentralized manner. Geographically, VISNs consist of catchment areas of their constituent medical centers (which, in turn, can be broken down into discrete counties).^13^ We used Veterans’ county of primary residence on the day of their appointment to identify the VISN in which they reside.

We categorized stop codes into 3 categories of care: primary care, mental health, and all other specialties. Our unit of analysis was the VISN–stop code–care setting (VHA or community care). eTable 1 in the Supplement contains a list of included stop codes, their assigned care category, national-level referral volumes, and mean appointment wait times.

### Statistical Analysis

Data analysis was performed from February to June 2022. Our analysis proceeded in 4 steps. We first estimated unadjusted mean and median appointment wait times for each combination of care category, care setting, and VISN. Observations were weighted by their number of consultations.

Second, we estimated ordinary least squares regression models to account for variations in specialty mix. We modeled wait times as our outcome with fixed effects for individual stop codes and VISN. Additionally, these fixed effects were interacted with a dummy variable indicating whether the appointment was for a VHA or community-based clinician. Regressions were also weighted by the number of consultations.

Third, we obtained regression predictions to create an adjusted wait time for each VISN–care category–care setting combination while holding the distribution of stop codes fixed at the national average. This standardization ensures that any estimated differences in VISN-level wait times are not driven by differences in specialty mix.

Fourth, we ranked VISNs across each of the 3 appointment categories. Using the VA MISSION Act access standards as a benchmark, we examined the extent to which different VHA medical centers or community-based clinicians met these standards.

Finally, we compared differences in wait times between the VA and community care clinicians using 2-sided *t* tests. We used an α = .05 cutoff for statistical significance in all analyses. In sensitivity analysis, we calculated wait times through the end of 2019 (to exclude the COVID-19 period).

Analyses were conducted using R statistical software version 4.0.5 (R Project for Statistical Computing). Interested researchers may use our data to examine individual specialties; deidentified data sets and annotated analytic code are available within a Mendeley Data repository.^11^ Data will be updated with new wait times approximately quarterly.

## Results

Our final sample included 22 632 918 million appointments for 4 846 892 unique veterans (Table 1). Among the VHA enrollees in our sample, 3 150 827 (65.0%) were White, 3 744 522 (77.3%) were male, 3 909 115 (80.7%) were non-Hispanic, and 2 390 994 (49.3%) were married; the mean (SD) age was 61.6 (15.5) years. When examining unadjusted wait times, veterans waited a mean (SD) of 27.9 (31.8) days for primary care, 34.6 (34.6) days for mental health care, and 35.9 (42.5) days for other specialties in the VHA. For care in the community, veterans waited a mean (SD) of 34.8 (41.6) days for primary care, 40.4 (42.1) days for mental health care, and 40.6 (42.3) days for other specialties. There was similar variation in appointment approval wait times to see a clinician (eTable 2 in the Supplement). These administrative delays were generally consistent over time (eFigure 1 in the Supplement).

**Table 1.** Characteristics of Veterans in the Study Sample^a^

Characteristic	Veterans, No. (%)		
	Overall (n = 4 846 892)	Non-VHA users (n = 3 042 060)	VHA users (n = 4 016 156)
Primary race			
Asian or Pacific Islander	95 151 (2.0)	60 099 (2.0)	79 163 (2.0)
Black	800 951 (16.5)	478 367 (15.7)	701 191 (17.5)
Native American	48 293 (1.0)	30 774 (1.0)	37 048 (0.9)
White	3 150 827 (65.0)	1 991 435 (65.5)	2 571 464 (64.0)
Unknown	751 670 (15.5)	481 384 (15.8)	627 290 (15.6)
Gender			
Female	618 288 (12.8)	375 282 (12.3)	447 201 (11.1)
Male	3 744 522 (77.3)	2 362 812 (77.7)	3 167 434 (78.9)
Unknown	484 082 (10.0)	303 966 (10.0)	401 521 (10.0)
Ethnicity			
Hispanic or Latino	305 194 (6.3)	190 161 (6.3)	256 294 (6.4)
Not Hispanic or Latino	3 909 115 (80.7)	2 447 038 (80.4)	3 225 888 (80.3)
Unknown	632 583 (13.1)	404 861 (13.3)	533 974 (13.3)
Marital status			
Married	2 390 994 (49.3)	1 514 596 (49.8)	1 944 655 (48.4)
Not married	1 927 229 (39.8)	1 190 772 (39.1)	1 628 637 (40.6)
Unknown	528 669 (10.9)	336 692 (11.1)	1 628 637 (11.0)
Age, mean (SD), y	61.6 (15.5)	61.9 (15.2)	61.6 (15.3)

Abbreviation: VHA, Veterans Health Administration.

^a^ Self-reported demographic characteristics of veterans in our sample were identified as of June 2021. Data were obtained from the VHA’s Corporate Data Warehouse.

Within VISNs, positive correlations were observed between wait times for the 3 care types. Among VHA appointments, correlation coefficients were *r* = 0.29 for primary care and other specialties, *r* = 0.58 for mental health and other specialties, and *r* = 0.16 for primary care and mental health. Correlation coefficients were higher for community-based appointments: *r* = 0.50 for primary care and other specialties, *r* = 0.73 for mental health and other specialties, and *r* = 0.43 for primary care and mental health. Finally, as with prior work,^11,12^ we found positive correlations between VA and community care wait times: *r* = 0.47 for other specialties and *r* = 0.41 for mental health. However, we found a negative correlation for primary care between VHA and community-based appointments (*r* = −0.27).

Nationally, no VISN met wait time standards on average for any care category. Across individual appointments, those being provided outside the VHA were less likely to meet access standards than those in the VHA. Although 49.9% of appointments with non-VHA clinicians met access standards, 44.2% of appointments with VHA clinicians exceeded wait time standards.

Unadjusted differences in wait times between VHA and non-VHA for each care category within VISN were all significant with *P* < .001 for all comparisons with 3 exceptions. For other specialties, there was no significant difference between VHA and non-VHA wait times in VISN 1 (*P* = .46); for mental health care, VISNs 1 and 2 had significant differences that were slightly less precise (*P* = .005 and *P* = .004, respectively). In the next sections, we describe adjusted means and SDs of VISN-level appointment and approval wait times for each of the 3 care types.

### Primary Care

There were a total of 606 086 primary care appointments. Among 385 264 appointments with VHA clinicians, the mean (SD) VISN-level appointment wait time was 29.0 (5.5) days for primary care. This ranged from a low of 22.4 (23.3) days in VISN 10, which covers Ohio, Indiana, and Michigan, to a high of 43.4 (49.6) days in VISN 15, which covers Kansas and Missouri (Table 2 and eFigure 2 in the Supplement). Slightly more than half (52.9%) of these appointments met the VHA wait time access standard of 20 days of less. Approval wait times were a mean (SD) of 1.6 (5.6) days, ranging from a low of 1.1 (1.0) days in VISN 10 to a high of 2.3 (3.1) days in VISN 1, which covers Connecticut, Massachusetts, Rhode Island, New Hampshire, Vermont, and Maine.

**Table 2.** Mean Appointment Wait Times and Referral Volumes, Primary Care^a^

Veterans Integrated Service Network	Wait time, mean (SD), d		Consultations (thousands), No.	
	Community care	VHA	Community care	VHA
01: Connecticut, Massachusetts, Rhode Island, New Hampshire, Vermont, Maine	50.2 (48.5)	28.3 (30.3)	2.8	34.2
02: New York, New Jersey	33.3 (34.6)	26.3 (25.8)	0.8	12.7
04: Pennsylvania, Delaware	44.6 (37.4)	28.2 (28.9)	1.0	17.6
05: Maryland, District of Columbia, West Virginia	49.1 (59.7)	27.0 (25.8)	1.3	3.9
06: Virginia, North Carolina	47.5 (40.6)	26.9 (31.7)	18.6	37.3
07: Alabama, Georgia, South Carolina	44.9 (40.1)	27.0 (29.1)	7.6	17.2
08: Florida, Puerto Rico, Virgin Islands	35.5 (37.1)	39.7 (43.9)	1.4	34.5
09: Kentucky, Tennessee	46.8 (37.2)	29.2 (25.7)	14.3	7.7
10: Ohio, Indiana, Michigan	39.1 (41.8)	22.4 (23.3)	11.3	69.8
12: Wisconsin, Illinois	52.4 (53.7)	22.5 (23.1)	6.1	17.8
15: Kansas, Missouri	32.9 (34.0)	43.4 (49.6)	8.0	6.9
16: Arkansas, Mississippi, Louisiana	35.6 (36.8)	33.6 (36.1)	29.9	12.2
17: Texas	26.2 (27.5)	29.4 (24.8)	24.1	27.6
19: Montana, Wyoming, Utah, Colorado	34.0 (36.3)	30.7 (35.9)	9.8	13.3
20: Washington, Oregon, Idaho, Alaska	34.6 (35.7)	29.1 (28.3)	50.2	24.0
21: California, Nevada, Hawaii, Philippines, Guam, American Samoa	34.5 (38.7)	23.6 (26.6)	4.2	15.1
22: California, Arizona, New Mexico	33.0 (28.3)	31.5 (28.8)	16.2	23.2
23: North Dakota, Minnesota, South Dakota, Nebraska, Iowa	25.4 (33.7)	23.9 (27.2)	13.2	10.1

Abbreviation: VHA, Veterans Health Administration.

^a^ The table presents regression-adjusted estimates of mean appointment wait times, controlling for regional differences in stop code mix. Primary states served are listed for each Veterans Integrated Service Network. Data were obtained from the VHA’s Corporate Data Warehouse.

Among 220 822 non-VA appointments, the mean (SD) VISN-level appointment wait time was 38.9 (8.2) days for primary care. This ranged from a low of 25.4 (33.7) days in VISN 23, which covers North and South Dakota, Minnesota, Nebraska, and Iowa, to a high of 52.4 (53.7) days in VISN 12, which covers Wisconsin and Illinois. Slightly less than half (46.8%) of these appointments met VHA wait time standards. The mean (SD) approval wait times were 2.5 (8.4) days overall, ranging from a low of 1.2 (1.2) days in VISN 23 to a high of 6.9 (2.3) days in VISN 1.

On average, 15 of the 18 VISNs (comprising 82% of total appointment volume) had shorter wait times for VHA vs community care. Additional descriptive statistics including median wait times and approval times are available in eTable 3 in the Supplement.

### Mental Health

There were a total of 795 444 mental health appointments. Among 588 503 appointments with VHA clinicians, the mean (SD) VISN-level appointment wait time was 33.6 (4.6) days for mental health. This ranged from a low of 24.7 (23.1) days in VISN 12, which covers Wisconsin and Illinois, to a high of 42.0 (37.9) days in VISN 6, which covers Virginia and North Carolina (Table 3 and eFigure 3 in the Supplement). We found that 41.1% of these appointments met the VHA wait time standard of 20 days or less. Approval wait times were a mean (SD) of 1.6 (6.7) days overall and ranged from a low of 1.2 (4.2) days in VISN 12 to a high of 2.7 (9.8) days in VISN 23.

**Table 3.** Mean Appointment Wait Times and Referral Volumes, Mental Health^a^

Veterans Integrated Service Network	Wait time, mean (SD), d		Consultations (thousands), No.	
	Community care	VHA	Community care	VHA
01: Connecticut, Massachusetts, Rhode Island, New Hampshire, Vermont, Maine	33.3 (41.6)	30.5 (33.9)	4.7	34.7
02: New York, New Jersey	29.3 (35.4)	30.1 (29.8)	2.5	22.3
04: Pennsylvania, Delaware	34.2 (38.1)	28.4 (31.1)	2.7	21.6
05: Maryland, District of Columbia, West Virginia	65.7 (71.5)	40.9 (36.8)	5.9	16.8
06: Virginia, North Carolina	55.0 (47.6)	42.0 (37.9)	12.4	51.3
07: Alabama, Georgia, South Carolina	52.0 (47.1)	35.8 (31.7)	13.7	42.2
08: Florida, Puerto Rico, Virgin Islands	44.6 (48.3)	34.3 (32.0)	16.1	60.8
09: Kentucky, Tennessee	44.7 (37.6)	34.7 (29.9)	7.3	27.9
10: Ohio, Indiana, Michigan	42.8 (41.4)	29.5 (27.1)	8.0	42.3
12: Wisconsin, Illinois	54.7 (51.0)	24.7 (23.1)	2.8	25.4
15: Kansas, Missouri	37.8 (32.7)	33.2 (29.6)	7.6	20.4
16: Arkansas, Mississippi, Louisiana	40.4 (45.7)	32.4 (29.7)	11.8	34.4
17: Texas	38.3 (34.1)	30.8 (25.6)	13.8	38.0
19: Montana, Wyoming, Utah, Colorado	47.2 (45.9)	34.7 (34.0)	17.0	30.7
20: Washington, Oregon, Idaho, Alaska	41.6 (35.1)	31.7 (31.9)	22.1	17.6
21: California, Nevada, Hawaii, Philippines, Guam, American Samoa	46.6 (43.4)	33.6 (31.3)	14.3	25.2
22: California, Arizona, New Mexico	35.2 (27.8)	41.2 (36.9)	39.5	50.7
23: North Dakota, Minnesota, South Dakota, Nebraska, Iowa	46.5 (43.9)	36.2 (45.8)	4.9	26.1

Abbreviation: VHA, Veterans Health Administration.

^a^ The table presents regression-adjusted estimates of mean appointment wait times, controlling for regional differences in stop code mix. Primary states served are listed for each Veterans Integrated Service Network. Data were obtained from the VHA’s Corporate Data Warehouse.

Among 206 941 non-VHA appointments, the mean (SD) VISN-level appointment wait time was 43.9 (9.0) days for mental health. This ranged from a low of 29.3 (35.4) days in VISN 2, which covers New York and New Jersey, to a high of 65.7 (71.5) days in VISN 5, which covers Maryland, Washington DC, and West Virginia. Only 35.6% of these appointments met VHA access standards. Approval wait times were a mean (SD) of 5.0 (14.5) days overall, ranging from a low of 2.3 (7.0) days in VISN 4, which covers Pennsylvania and Delaware, to a high of 11.4 (29.3) days in VISN 19, which covers Montana, Wyoming, Utah, and Colorado.

On average, 16 of the 18 VISNs (comprising 88% of total appointment volume) had shorter wait times for VHA clinicians vs community care. Additional descriptive statistics including median wait times and approval times are available in eTable 4 in the Supplement.

### All Other Specialties

There were a total of 21 231 388 appointments for all other specialties. Among 12 038 000 appointments with VHA clinicians, the mean (SD) VISN-level appointment wait time was 35.4 (2.7) days for all other specialties. This ranged from a low of 30.3 (31.5) days in VISN 2 to a high of 41.9 (42.4) days in VISN 6 (Table 4 and Figure). Approval wait times were 2.0 (11.5) days overall and ranged from a low of 1.3 (7.1) days in VISN 16, which covers Arkansas, Mississippi, and Louisiana, to a high of 3.0 (11.5) days in VISN 20, which covers Washington, Oregon, Idaho, and Alaska.

**Table 4.** Mean Appointment Wait Times and Referral Volumes, All Other Specialties^a^

Veterans Integrated Service Network	Wait time, mean (SD), d		Consultations (thousands), No.	
	Community care	VHA	Community care	VHA
01: Connecticut, Massachusetts, Rhode Island, New Hampshire, Vermont, Maine	36.8 (36.5)	35.5 (39.5)	306.2	515.8
02: New York, New Jersey	38.7 (36.1)	30.3 (31.5)	156.0	533.3
04: Pennsylvania, Delaware	37.5 (37.5)	34.6 (35.5)	346.7	468.4
05: Maryland, District of Columbia, West Virginia	49.6 (50.8)	35.0 (34.7)	221.1	345.1
06: Virginia, North Carolina	53.1 (47.9)	41.9 (42.4)	563.2	831.4
07: Alabama, Georgia, South Carolina	54.8 (48.9)	35.9 (36.3)	642.2	729.9
08: Florida, Puerto Rico, Virgin Islands	43.8 (42.5)	38.7 (40.4)	488.0	1208.9
09: Kentucky, Tennessee	44.4 (38.6)	37.2 (34.3)	471.7	498.3
10: Ohio, Indiana, Michigan	40.8 (38.2)	30.9 (31.7)	480.4	1134.6
12: Wisconsin, Illinois	41.0 (40.7)	34.8 (36.6)	354.1	521.4
15: Kansas, Missouri	34.7 (31.6)	33.8 (34.6)	425.0	482.9
16: Arkansas, Mississippi, Louisiana	39.8 (36.7)	35.1 (37.0)	685.2	817.3
17: Texas	36.1 (34.3)	35.2 (35.3)	855.2	750.1
19: Montana, Wyoming, Utah, Colorado	44.6 (40.8)	37.6 (38.6)	717.5	590.8
20: Washington, Oregon, Idaho, Alaska	42.6 (39.5)	35.8 (37.6)	688.5	502.9
21: California, Nevada, Hawaii, Philippines, Guam, American Samoa	42.5 (38.3)	33.8 (32.9)	489.0	684.7
22: California, Arizona, New Mexico	36.5 (29.8)	38.2 (37.3)	675.1	934.9
23: North Dakota, Minnesota, South Dakota, Nebraska, Iowa	35.9 (35.4)	33.5 (37.9)	628.1	487.2

Abbreviation: VHA, Veterans Health Administration.

^a^ The table presents regression-adjusted estimates of mean appointment wait times, controlling for regional differences in stop code mix. Primary states served are listed for each Veterans Integrated Service Network. Data were obtained from the VHA’s Corporate Data Warehouse.

**Figure.**
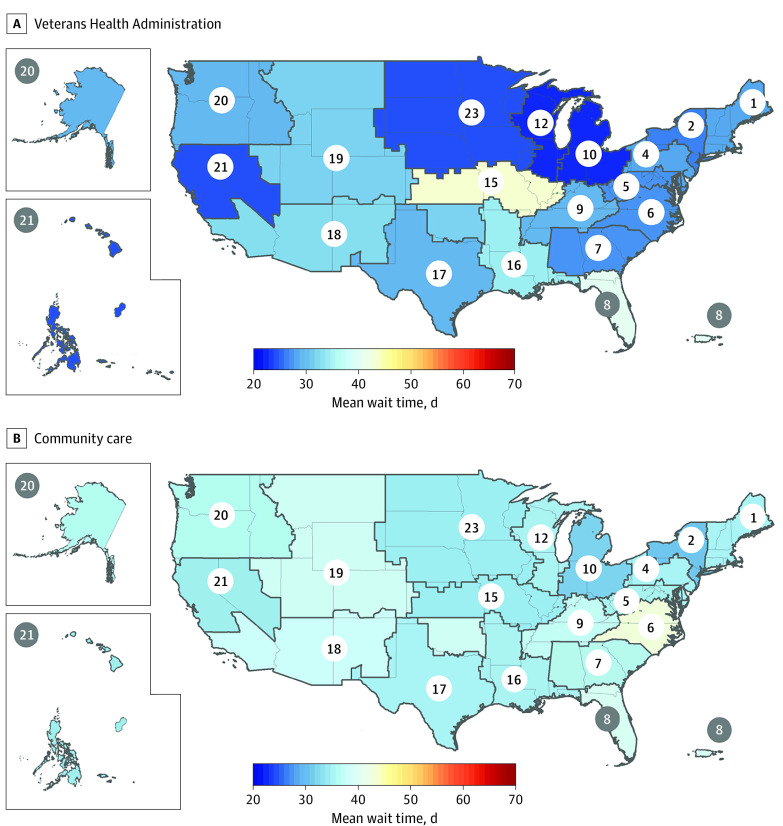
Mean Appointment Wait Times for All Other Specialties, by Veterans Integrated Service Network The circled numbers indicate Veterans Integrated Services Networks, which are regional networks of Veterans Health Administration medical centers. Veterans Integrated Services Network 8 includes Puerto Rico and the Virgin Islands. VISN 21 includes Hawaii, Guam, American Samoa, and Philippines. The figure presents regression-adjusted estimates of mean appointment wait times, controlling for regional differences in stop code mix. Data were obtained from the Veterans Health Administration Corporate Data Warehouse.

Among 9 193 388 non-VA appointments, the mean (SD) VISN-level appointment wait time was 41.9 (5.9) days for all other specialties. This ranged from a low of 34.7 (31.6) days in VISN 15 to a high of 54.8 (48.9) days in VISN 7, which covers Alabama, Georgia, and South Carolina. Approximately half (50.6%) of these appointments met the VHA’s wait time standard of 28 days or less. Approval wait times were 3.9 (12.3) days overall and ranged from a low of 2.7 (8.5) days in VISN 12 to a high of 5.9 (10.2) days in VISN 1.

On average, 17 of the 18 VISNs (comprising 92% of total appointment volume) had shorter wait times for VHA clinicians vs community care. Additional descriptive statistics including median wait times and approval times are available in eTable 5 in the Supplement.

Finally, eTable 6 in the Supplement presents appointment wait times excluding the COVID-19 years (2020 and 2021). In general, wait times were shorter before the onset of the COVID-19 pandemic.

## Discussion

In this cross-sectional study, we leveraged a novel data resource and provided the first national analysis of the wait times that veterans experience when seeking medical care within the VHA vs community care. We observed substantial geographic variation within all 3 appointment categories, as well as variation in wait times between VHA and private sector clinicians. The VHA outperformed community care in terms of wait times for a majority of VISNs; these results suggest that policies to liberalize veterans’ access to private-sector clinicians may not lead to lower wait times in those regions.^12,14^

Our results also indicate that a sizeable proportion of veterans experienced wait times that exceeded the VHA access standards. Overall, 44.2% of appointments with VHA clinicians exceeded wait time standards, and 49.9% of appointments with community care clinicians exceeded wait time standards. Although the Optum and TriWest networks include nearly 1 million clinicians, future research should examine whether these networks are adequate to meet demand from VHA enrollees across all specialties in a timely manner. The VHA should also consider expansion of its ongoing initiatives to enhance veterans’ access to care in underserved areas. The VA Office of Rural Health supports programs that address the health care needs of rural veterans through telemedicine, social workers, and transportation services. For instance, VA Clinical Resource Hubs provide primary, mental health, and specialty care to rural areas through a variety of modalities intended to improve access to care. Finally, more work is needed to understand whether disparities in wait times are associated with patient-level characteristics such as race and ethnicity, gender, age, or health status. Our findings comport with prior research indicating that primary care, mental health, and other specialty medical professionals are not well distributed with respect to underlying need.^8,15,16^

### Limitations

Our findings should be considered in light of several limitations. First, our data do not include clinician identifiers, and we are unable to determine the extent to which the clinicians offering care to veterans in the community are representative of clinicians more broadly. However, we believe our large sample size, nationwide reach, and the size of the Optum and TriWest clinician networks represent a marked improvement in the measurement of US appointment wait times. Second, our data do not allow us to distinguish the sources of geographic variation in wait times or whether the wait times experienced by veterans were clinically appropriate. Third, our results are based on the experiences of veterans who seek medical care in the private sector and may not generalize to the US general population. For example, wait times experienced by veterans might be different if private sector medical professionals limit appointments for these patients because of their lower average health status compared with the general population^17^ or because of the VHA’s Medicare reimbursement rate,^7^ which are lower than those offered by private insurers. On the other hand, veterans, who often have alternative sources of coverage,^18^ might use their VHA health benefits selectively, and these patterns may not comport with the general population. Fourth, because of the large number of specialties included in our analyses, we were unable to describe our findings for individual specialties. However, we have made our full data and analytic code available for researchers who may wish to examine these results. Fifth, our sample sizes for primary care and mental health appointments are much smaller than our sample for all other specialties, since these services are primarily delivered within the VHA system. Veterans who obtain these services through community care may not be representative of the larger population of VHA enrollees.

## Conclusions

In conclusion, we developed a large publicly accessible database of primary and specialty care appointment wait times based on veterans’ experience and identified substantial geographic variation across the US. Our findings imply that areas with high-wait times for community care are not expected to benefit from liberalized access under the Veterans Access, Choice and Accountability Act in 2014 and the VA MISSION Act in 2018. Policies and interventions, such as physician relocation incentives, telehealth, or mobile deployment units, may be needed to increase the number of ways patients in underserved areas can interact with the health care system.

## Notes

Supplement. **eTable 1.** List of Included Stop Codes **eTable 2.** Summary Statistics for Appointment and Approval Wait Times, by Care Category **eFigure 1.** Trends in Approval Wait Times, by Care Category and Setting **eFigure 2.** Mean Appointment Wait Times for Primary Care, by Veterans Integrated Service Network (VISN) **eTable 3.** Median Appointment Wait Times, Mean Approval Times, and Referral Volumes for Primary Care **eFigure 3.** Mean Appointment Wait Times for Mental Health, by Veterans Integrated Service Network (VISN) **eTable 4.** Median Appointment Wait Times, Mean Approval Times, and Referral Volumes for Mental Health **eTable 5.** Median Appointment Wait Times, Mean Approval Times, and Referral Volumes for All Other Specialties **eTable 6.** Mean Appointment Wait Times and Referral Volumes, 2018-2019
