# Errors in the Figure

**DOI:** 10.1001/jamanetworkopen.2022.50857

**Published:** 2022-12-22

In the Original Investigation titled “Geographic Variation in Appointment Wait Times for US Military Veterans,”^1^ published August 25, 2022, there were errors in the colors shown in the Figure. This article has been corrected.^1^
