# Supplementary material for: Geographic Variation in Appointment Wait Times for US Military Veterans
Source: JAMA Netw Open. 2022 Aug 25;5(8):e2228783. doi: 10.1001/jamanetworkopen.2022.28783 (PMC9412224; doi:10.1001/jamanetworkopen.2022.28783)
Supplement: Supplement. — eTable 1. List of Included Stop Codes eTable 2. Summary Statistics for Appointment and Approval Wait Times, by Care Category eFigure 1. Trends in Approval Wait Times, by Care Category and Setting eFigure 2. Mean Appointment Wait Times for Primary Care, by Veterans Integrated Service Network (VISN) eTable 3. Median Appointment Wait Times, Mean Approval Times, and Referral Volumes for Primary Care eFigure 3. Mean Appointment Wait Times for Mental Health, by Veterans Integrated Service Network (VISN) eTable 4. Median Appointment Wait Times, Mean Approval Times, and Referral Volumes for Mental Health eTable 5. Median Appointment Wait Times, Mean Approval Times, and Referral Volumes for All Other Specialties eTable 6. Mean Appointment Wait Times and Referral Volumes, 2018-2019 [file jamanetwopen-e2228783-s001.pdf]

Supplemental Online Content

Feyman Y, Asfaw DA, Griffith KN. Geographic variation in appointment wait times for US military veterans. *JAMA Netw Open.* 2022;5(8):e2228783. doi:10.1001/jamanetworkopen.2022.28783

eTable 1. List of Included Stop Codes

eTable 2. Summary Statistics for Appointment and Approval Wait Times, by Care Category

eFigure 1. Trends in Approval Wait Times, by Care Category and Setting

eFigure 2. Mean Appointment Wait Times for Primary Care, by Veterans Integrated Service Network (VISN)

eTable 3. Median Appointment Wait Times, Mean Approval Times, and Referral Volumes for Primary Care

eFigure 3. Mean Appointment Wait Times for Mental Health, by Veterans Integrated Service Network (VISN)

eTable 4. Median Appointment Wait Times, Mean Approval Times, and Referral Volumes for Mental Health

eTable 5. Median Appointment Wait Times, Mean Approval Times, and Referral Volumes for All Other Specialties

eTable 6. Mean Appointment Wait Times and Referral Volumes, 2018-2019

This supplemental material has been provided by the authors to give readers additional information about their work.

© 2022 Feyman Y et al. *JAMA Network Open.*

eTable 1. List of Included Stop Codes

		Community care		Veterans Health Administration	
Stop code	Group name	Mean wait time (days)	Total # of consults	Mean wait time (days)	Total # of consults
**Primary Care**					
322	COMP WOMEN'S HLTH	39.4	3707	31.5	49621
301	GENERAL INTERNAL MEDICINE	11.7	15289	31.7	52075
318	GERI PROB CONSULT CLINIC	7.5	38416	41.9	17383
177	HBPC - OTHER	4.8	4987	23.3	1259
348	PRIMARY CARE SHARED APPT	52.8	1014	27.2	17879
323	PRIMARY CARE/MEDICINE	44.4	157409	25.5	247047
**Mental Health**					
502	MENTAL HEALTH CLINIC - IND	37.9	151700	32.3	371575
533	MH INTERVNTION BIOMED CARE IND	40.4	2491	34.4	17015
509	PSYCHIATRY	47.7	23348	31.5	55357
538	PSYCHOLOGICAL TESTING	80.5	3311	50.7	62716
510	PSYCHOLOGY	43.5	26091	34.9	81840
**All Other Specialties**					
190	ADULT DAY HEALTH CARE	14.6	4013	18.1	1813
302	ALLERGY IMMUNOLOGY	48.7	76507	35.8	28302
392	AMB ECG MONITORING	17.1	1800	21.9	75622
317	ANTI-COAGULATION CLINIC	18.2	1713	12.7	22644
203	AUDIOLOGY	48.7	120115	43.9	89461
487	BARIATRIC SURG	56.0	1950	35.2	2922
333	CARDIAC CATHETERIZATION	25.6	4159	24.7	46922
391	CARDIAC ECHO	36.0	18229	39.2	343838
334	CARDIAC STRESS TEST	36.1	6873	28.9	142601
402	CARDIAC SURGERY	25.9	6247	21.4	4912
231	CARDIO-PULM REHAB	37.5	32625	33.7	10496
303	CARDIOLOGY	40.2	373989	34.2	628891
436	CHIROPRACTIC CARE	30.7	406410	36.1	104248
311	CIED DEVICES	19.9	2969	36.5	55883
159	CIH TREATMENT	32.2	213267	36.4	54422
160	CLINICAL PHARMACY	30.5	1017	23.9	136996
150	COMPUTERIZED TOMOGRAPHY (CT)	30.3	129669	39.6	10079
180	DENTAL	41.6	583811	30.8	134268
304	DERMATOLOGY	50.5	404601	30.9	553065
106	EEG	44.0	4226	28.4	35621
130	EMERGENCY DEPT	1.9	54444	29.7	18854
212	EMG - ELECTROMYOGRAM	53.2	46867	40.7	181699
305	ENDOCRINOLOGY	66.5	54173	36.8	129252
369	EP LAB	43.3	6789	28.4	21084
307	GASTROENTEROLOGY	54.7	259918	49.7	402289

© 2022 Feyman Y et al. *JAMA Network Open.*

401	GENERAL SURGERY	32.5	105683	28.0	326088
340	GENOMIC CARE	77.9	5036	50.9	6575
321	GI ENDOSCOPY	59.0	234623	67.1	672063
404	GYNECOLOGY	51.0	46422	31.6	74377
405	HAND SURGERY	40.9	6132	34.4	89232
680	HCBC ASSESSMENT	7.9	106801	12.4	6606
139	HEALTH/WELLBEING SRVS	44.2	9241	26.8	38742
308	HEMATOLOGY	32.8	54220	24.6	75539
337	HEPATOLOGY CLINIC	56.6	3732	42.2	99412
118	HOME TREATMENT SERVICES	13.0	148166	21.2	18290
351	HOSPICE CARE	6.3	21927	15.9	8273
310	INFECTIOUS DISEASE	28.7	12549	23.1	42146
110	INTERVENT RAD CLINIC (IR)	27.8	7836	11.9	5769
153	INTERVENT RAD PROCEDURE (IR)	24.4	28842	17.1	44337
108	LABORATORY	15.0	67683	20.9	4612
151	MAGNETIC RESONANCE IMAGING/MRI	30.0	309094	32.6	56239
703	MAMMOGRAM (MG)	48.4	287192	42.4	9632
329	MEDICAL PROCEDURE UNIT	9.9	10716	38.0	46804
145	MYOCARD PERF STUDIES	43.5	1107	22.3	3215
315	NEUROLOGY	66.3	192367	44.5	285489
406	NEUROSURGERY	46.0	174327	31.3	89633
109	NUC MED & PET (NM & PET)	27.3	62953	27.9	32369
117	NURSING (2ND ONLY)	11.5	5124	27.2	29626
123	NUTRITION/DIETETICS-INDIVIDUAL	45.0	1958	23.1	117413
339	OBSTETRICS	23.6	24735	40.0	1665
206	OCCUPATIONAL THERAPY	29.0	45655	23.0	375449
316	ONCOLOGY/TUMOR	29.7	80587	22.8	88130
407	OPHTHALMOLOGY	49.9	494016	43.1	185215
523	OPIOID TREATMENT PROGRAM	17.7	1173	22.7	5159
408	OPTOMETRY	45.8	622804	44.1	148918
409	ORTHO/JOINT SURG	39.5	470973	32.5	560916
403	OTOLARYNGOLOGY/ENT	44.8	159663	30.0	332552
420	PAIN CLINIC	45.1	279214	34.8	246825
353	PALLIATIVE CARE	9.0	2025	22.4	21221
429	PATIENT CARE IN OR	44.2	3308	50.6	32482
205	PHYSICAL THERAPY	31.3	649014	25.5	960809
410	PLASTIC SURGERY	43.3	18597	30.8	113956
240	PM&R ASSIST TECH CLINIC	46.6	1619	24.5	3083
201	PM&RS PHYSICIAN	36.3	43566	33.9	356715
411	PODIATRY	43.3	238850	36.6	383796
146	POSITRON EMISSION TOMOGRAPHY (PET)	28.8	2784	28.5	2451
104	PULMONARY FUNCTION	47.8	19913	41.7	331179
312	PULMONARY/CHEST	54.6	103715	36.4	242158

© 2022 Feyman Y et al. *JAMA Network Open.*

149	RADIATION ONCOLOGY	21.3	81790	14.1	26593
250	REHAB SRVCS GROUP	36.0	20725	26.7	29936
313	RENAL/NEPHROL(EXCEPT DIALYSIS)	49.0	91763	36.0	80615
116	RESPIRATORY THERAPY	42.5	3674	33.2	187024
314	RHEUMATOLOGY/ARTHRITIS	75.8	36834	34.8	99487
586	RRTP INDIVIDUAL	8.6	1237	27.8	2572
349	SLEEP MEDICINE	60.7	77217	39.4	429070
143	SLEEP STUDY	46.6	126857	43.1	146412
125	SOCIAL WORK SERVICE	9.7	11506	22.8	59237
204	SPEECH-LANGUAGE PATHOLOGY	35.4	15008	19.9	115950
489	SPINAL SURG	44.5	9014	36.0	6537
513	SUBSTANCE USE DISORDER IND	10.9	2513	21.4	59838
488	SURG ONCOLOGY	25.9	1683	20.6	2447
435	SURGICAL PROCEDURE UNIT	24.6	4336	33.2	85723
428	TELEPHONE/OPTOMETRY	44.7	14448	52.7	6880
425	TELEPHONE/PROSTHETICS/ORTHOTIC	56.9	2132	17.1	11951
413	THORACIC SURGERY	27.6	7241	20.8	25386
115	ULTRASOUND (US)	31.0	134777	41.3	72865
414	UROLOGY CLINIC	46.9	270573	35.1	352985
421	VASCULAR LABORATORY	40.4	17781	65.1	233755
415	VASCULAR SURGERY	37.8	71914	32.2	118600
704	WMS SPECIFIC PREVENTIVE CARE	48.9	3749	38.8	10845
142	WOUND TREAT & OSTOMY CARE	16.5	22775	21.4	47722
105	X-RAY & FLUORO (XR & RF)	22.8	181518	23.8	14226

Source: Veterans Health Administration’s Corporate Data Warehouse.
Notes: Stop codes were included if they had at least 1,000 consults each for VHA and community-based providers from January 2018 through June 2021.

eTable 2. Summary Statistics for Appointment and Approval Wait Times, by Care Category

VETERANS HEALTH ADMINISTRATION							
**Appointment Wait Times**	**Mean**	**Median**	**SD**	**IQR**	**Min**	**Max**	**Number of Consults**
Primary Care	27.9	19.0	31.8	26.0	0.0	963	385264
Mental Health Care	34.6	25.0	34.6	32.0	0.0	783	588503
Other Specialties Care	35.9	25.0	42.5	32.0	0.0	1378	12038000
**Approval Wait Times**							
Primary Care	1.6	0.0	5.6	1.0	0.0	540	385264
Mental Health Care	1.6	0.0	6.7	1.0	0.0	537	588503
Other Specialties Care	2.0	0.0	11.5	1.0	0.0	1360	12038000
**COMMUNITY CARE**							
**Appointment Wait Times**	**Mean**	**Median**	**SD**	**IQR**	**Min**	**Max**	**Number of Consults**
Primary Care	34.8	22.0	41.6	41.0	0.0	560	220822
Mental Health Care	40.4	28.0	42.1	37.0	0.0	559	206941
Other Specialties Care	40.6	28.0	42.3	38.0	0.0	893	9193388
**Approval Wait Times**							
Primary Care	2.5	1.0	8.4	3.0	0.0	664	220822
Mental Health Care	5.0	1.0	14.5	4.0	0.0	441	206941
Other Specialties Care	3.9	1.0	12.3	3.0	0.0	912	9193388

Source: Veterans Health Administration's Corporate Data Warehouse.
Notes: Wait times and approval times are unadjusted and calculated at the individual level across all study years (2018-2021). SD = standard deviation. IQR = interquartile range.

© 2022 Feyman Y et al. *JAMA Network Open.*

eFigure 1. Trends in Approval Wait Times, by Care Category and Setting

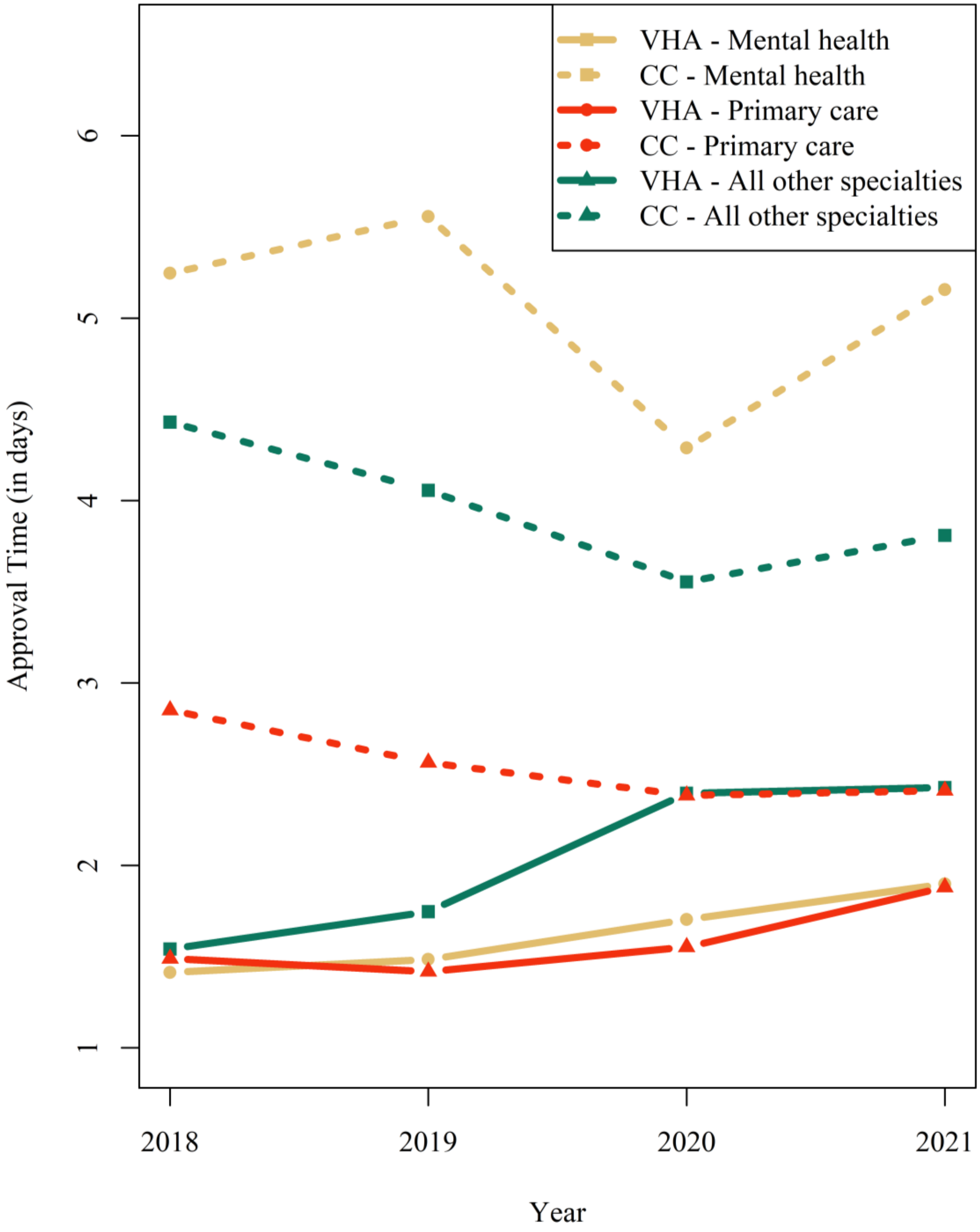

Source: Veterans Health Administration's Corporate Data Warehouse. **Notes:** Approval wait times were calculated as the difference (in days) between when the referral was initially made and when the referral was approved by the local medical center.

© 2022 Feyman Y et al. *JAMA Network Open.*

eFigure 2. Mean Appointment Wait Times for Primary Care, by Veterans Integrated Service Network (VISN)

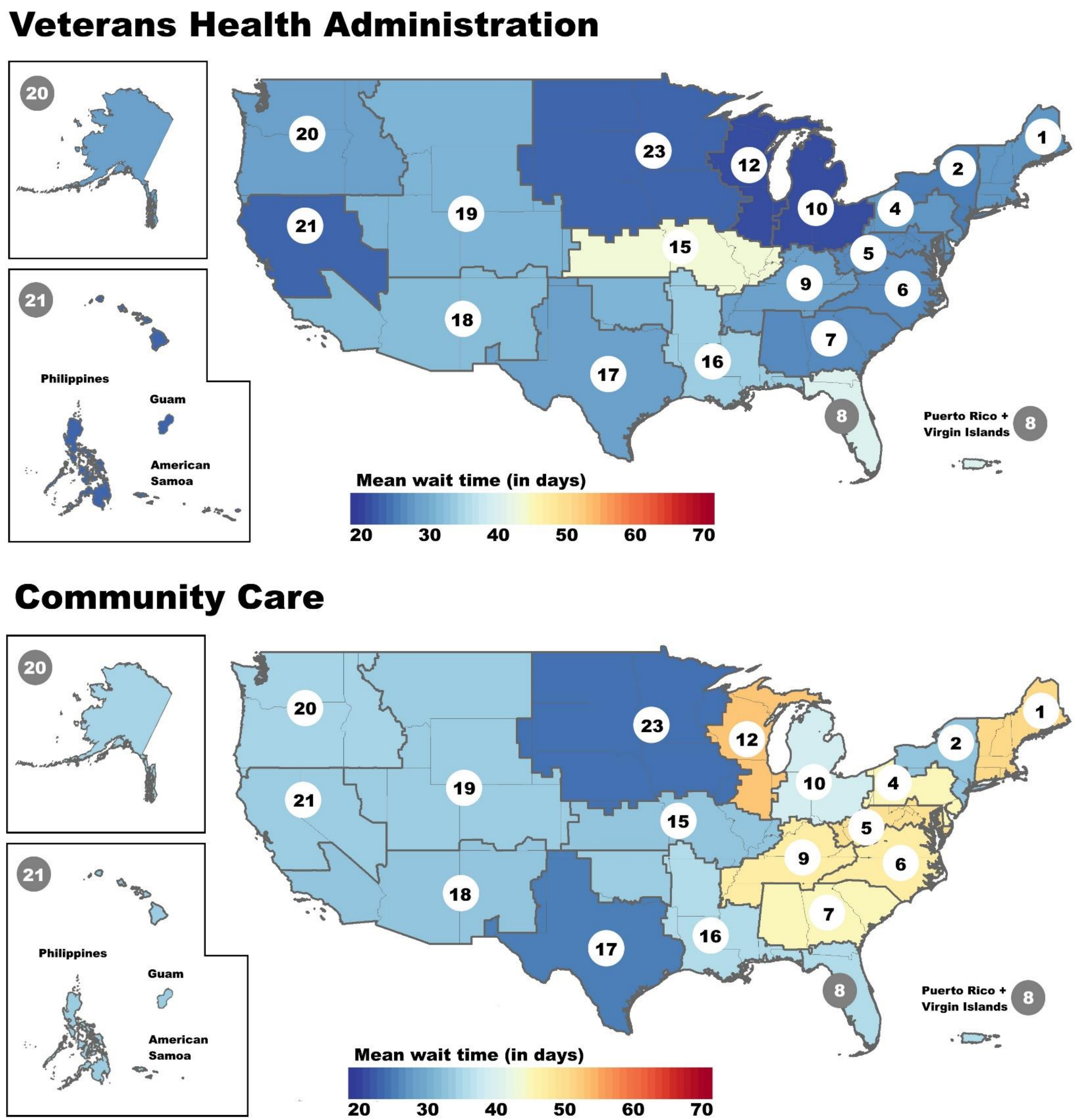

Source: Veterans Health Administration's Corporate Data Warehouse. **Notes:** The circled numbers indicate Veterans Integrated Services Networks (VISNs), which are regional networks of VHA medical centers. The figure presents regression-adjusted estimates of mean appointment wait times, controlling for regional differences in stop code mix.

© 2022 Feyman Y et al. *JAMA Network Open.*

eTable 3. Median Appointment Wait Times, Mean Approval Times, and Referral Volumes for Primary Care

	Community care			Veterans Health Administration		
**Veterans Integrated Service Network (VISN)**	**Median wait time**	**Mean approval time**	**Number of consults**	**Median wait time**	**Mean approval time**	**Number of consults**
**01: CT, MA, RI, NH, VT, ME**	36.3 (55.6)	6.9 (2.3)	2770	20.8 (22.7)	2.3 (3.1)	34185
**02: NY, NJ**	22.4 (32.1)	2.4 (1.6)	793	19.9 (24.5)	1.6 (0.8)	12695
**04: PA, DE**	35.8 (44.2)	1.3 (1.5)	983	20.7 (27)	1.5 (0.9)	17644
**05: MD, DC, WV**	30.4 (43.2)	2.5 (2.2)	1314	19 (28.1)	2.2 (0.8)	3942
**06: VA, NC**	36.6 (40.2)	4.3 (1.7)	18591	16.6 (24.3)	1.7 (2)	37269
**07: AL, GA, SC**	34.4 (39.7)	4.5 (1.6)	7647	18.8 (24.4)	1.6 (2.1)	17190
**08: FL, PR, VI**	24.8 (34.7)	2.1 (2.2)	1417	27 (34.6)	2.2 (0.6)	34534
**09: KY, TN**	41.1 (40.2)	3 (1.1)	14264	23.5 (24.4)	1.1 (0.9)	7746
**10: OH, IN, MI**	26.7 (35.9)	3.5 (1)	11274	16.4 (22.6)	1 (1)	69838
**12: WI, IL**	34.5 (53.7)	2 (1)	6076	15.9 (19)	1 (0)	17789
**15: KS, MO**	22.1 (32.1)	2.6 (1.6)	8005	29.5 (43.9)	1.6 (0.3)	6936
**16: AR, MS, LA**	24.5 (29.3)	3 (1.2)	29882	23.7 (30.7)	1.2 (1.4)	12193
**17: TX**	19.4 (20)	2.8 (1.8)	24128	23.7 (21.7)	1.8 (0.9)	27620
**19: MT, WY, UT, CO**	21.2 (32.9)	4.1 (1.6)	9781	20.8 (29.3)	1.6 (1.8)	13346
**20: WA, OR, ID, AK**	25.7 (31.3)	1.5 (1.6)	50232	22.2 (26)	1.6 (0.9)	23995
**21: CA, NV, HI, PH, GU, AS**	22.3 (31.5)	1.8 (1.5)	4234	16.2 (21.6)	1.5 (1)	15120
**22: CA, AZ, NM**	26.6 (27.5)	1.4 (1.9)	16220	24.9 (30.6)	1.9 (0.3)	23157
**23: ND, MN, SD, NE, IA**	14.3 (28.3)	1.2 (1.2)	13211	16.4 (23.5)	1.2 (0.1)	10065

Source: Veterans Health Administration's Corporate Data Warehouse. **Notes**: The table presents regression-adjusted estimates of median appointment wait times, controlling for regional differences in stop code mix. Standard deviations in parentheses. Primary states served are listed for each VISN. PH: Philippines; GU: Guam; AS: American Samoa.

© 2022 Feyman Y et al. *JAMA Network Open.*

eFigure 3. Mean Appointment Wait Times for Mental Health, by Veterans Integrated Service Network (VISN)

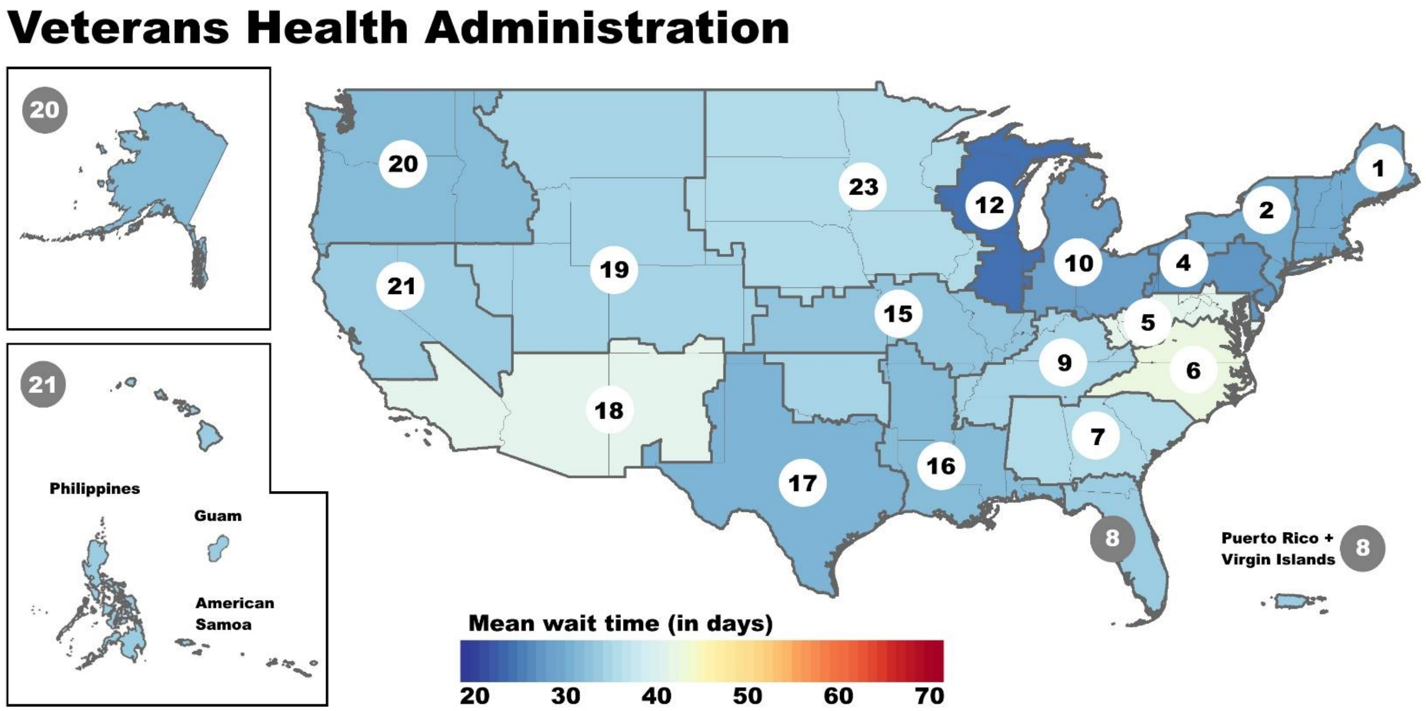

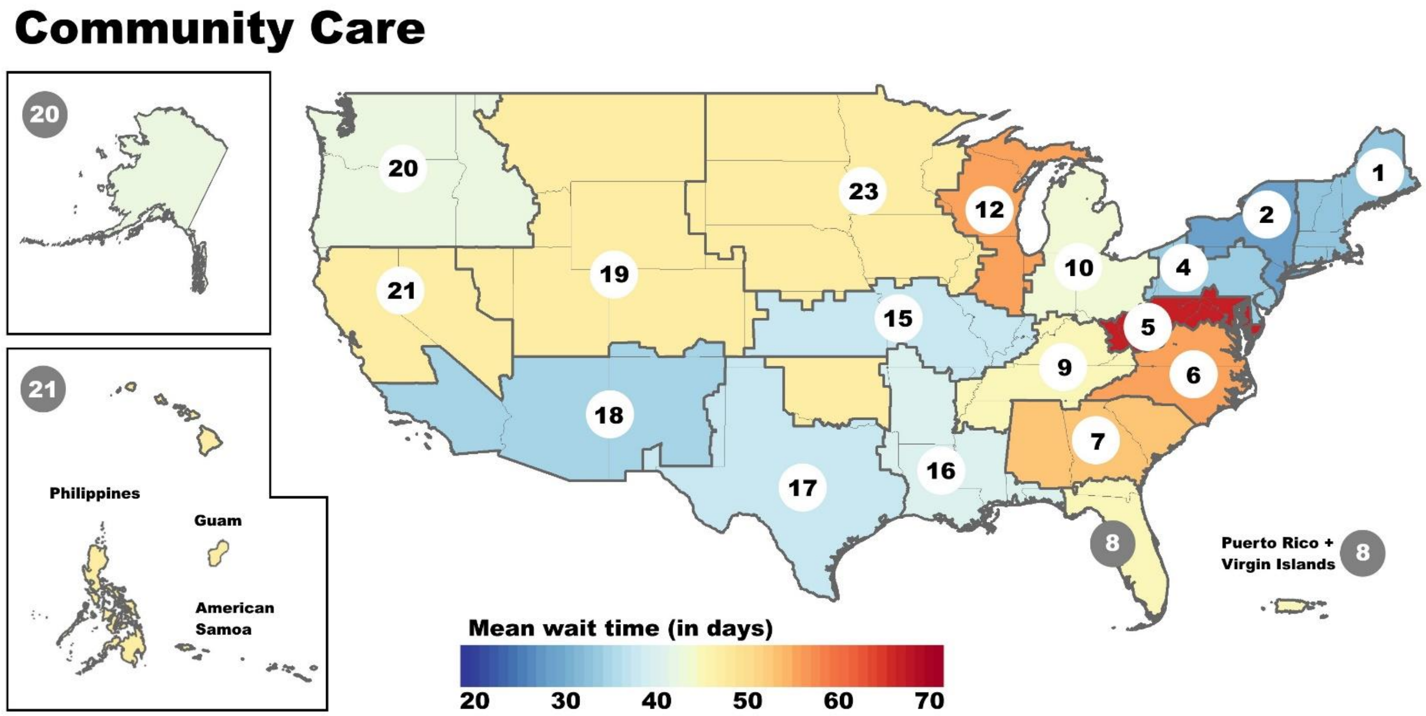

Source: Veterans Health Administration's Corporate Data Warehouse. **Notes:** The circled numbers indicate Veterans Integrated Services Networks (VISNs), which are regional networks of VHA medical centers. The figure presents regression-adjusted estimates of mean appointment wait times, controlling for regional differences in stop code mix.

© 2022 Feyman Y et al. *JAMA Network Open.*

eTable 4. Median Appointment Wait Times, Mean Approval Times, and Referral Volumes for Mental Health

	Community care			Veterans Health Administration		
**Veterans Integrated Service Network (VISN)**	**Median wait time**	**Mean approval time**	**Number of consults**	**Median wait time**	**Mean approval time**	**Number of consults**
**01: CT, MA, RI, NH, VT, ME**	21.1 (34.1)	6.8 (13.7)	4679	21.1 (27.1)	1.3 (5.9)	34740
**02: NY, NJ**	17.3 (31.6)	3.3 (8.7)	2529	21.5 (27.3)	1.6 (4.7)	22259
**04: PA, DE**	22.7 (42.5)	2.3 (7)	2678	21 (22.9)	1.6 (5.9)	21633
**05: MD, DC, WV**	43.8 (60.5)	5.5 (15.1)	5938	31.5 (38.7)	2.6 (7.7)	16815
**06: VA, NC**	42 (45.9)	7.4 (17.7)	12360	31.7 (37.4)	1.5 (8.1)	51334
**07: AL, GA, SC**	39.7 (56.7)	2.4 (7.2)	13713	27.5 (31.7)	1.7 (6.6)	42235
**08: FL, PR, VI**	28.1 (42.5)	5.5 (15.9)	16106	25.3 (32.2)	1.5 (6.5)	60772
**09: KY, TN**	38.1 (41.9)	4.4 (12.5)	7257	27.4 (30.6)	1.7 (5.3)	27898
**10: OH, IN, MI**	32.1 (39.1)	3.9 (9.4)	7952	22.4 (27.8)	1.3 (6.2)	42302
**12: WI, IL**	40.5 (51.9)	3 (9.9)	2767	17.9 (21.6)	1.2 (4.2)	25448
**15: KS, MO**	29.1 (37.7)	4.6 (8.6)	7631	25.3 (31.3)	1.4 (3.7)	20447
**16: AR, MS, LA**	26.2 (39.5)	3.7 (9.7)	11833	24.4 (29.2)	1.4 (7.7)	34363
**17: TX**	29.7 (35.6)	4.7 (11.1)	13752	25 (28.1)	1.4 (4.1)	37981
**19: MT, WY, UT, CO**	33.9 (42.4)	11.4 (29.3)	16959	24.6 (31.2)	1.7 (7.7)	30722
**20: WA, OR, ID, AK**	34.4 (35.7)	3 (12.5)	22110	22 (30.2)	2.1 (7.1)	17606
**21: CA, NV, HI, PH, GU, AS**	33.5 (41.9)	3.8 (12.3)	14289	24.9 (27.7)	1.5 (6.7)	25213
**22: CA, AZ, NM**	29.1 (28)	5.9 (11.4)	39462	31.1 (37.4)	1.7 (5.9)	50684
**23: ND, MN, SD, NE, IA**	33.9 (46.8)	3.8 (11.4)	4926	24.5 (32.2)	2.7 (9.8)	26051

Source: Veterans Health Administration's Corporate Data Warehouse. **Notes**: The table presents regression-adjusted estimates of median appointment wait times, controlling for regional differences in stop code mix. Standard deviations in parentheses. Primary states served are listed for each VISN. PH: Philippines; GU: Guam; AS: American Samoa.

© 2022 Feyman Y et al. *JAMA Network Open.*

eTable 5. Median Appointment Wait Times, Mean Approval Times, and Referral Volumes for All Other Specialties

	Community care			Veterans Health Administration		
**Veterans Integrated Service Network (VISN)**	**Median wait time**	**Mean approval time**	**Number of consults**	**Median wait time**	**Mean approval time**	**Number of consults**
**01: CT, MA, RI, NH, VT, ME**	26.8 (34.6)	5.9 (10.2)	306229	24.1 (31.2)	1.9 (7.5)	515780
**02: NY, NJ**	28.6 (35.3)	4.3 (11.3)	155960	22.2 (27.4)	2.1 (7.9)	533315
**04: PA, DE**	26.9 (34.9)	2.8 (9.8)	346667	24.6 (34)	2 (8)	468363
**05: MD, DC, WV**	34.6 (44.3)	5.7 (13.6)	221090	26 (33.2)	2.1 (7.3)	345147
**06: VA, NC**	39.2 (49)	5.5 (13.9)	563229	30.7 (39.2)	2.1 (9.9)	831437
**07: AL, GA, SC**	41 (47.5)	4.1 (11)	642230	26.3 (34.7)	2 (8.9)	729897
**08: FL, PR, VI**	31.4 (39.6)	4.5 (10.8)	487998	27.5 (36.3)	1.8 (7)	1208924
**09: KY, TN**	34.8 (37.6)	4.3 (11)	471730	28.4 (34.9)	1.8 (6)	498338
**10: OH, IN, MI**	29.9 (37.8)	4.5 (10.8)	480402	23.4 (28.6)	1.7 (7.9)	1134641
**12: WI, IL**	30.1 (36.8)	2.7 (8.5)	354064	25.2 (29.6)	2.1 (7.7)	521359
**15: KS, MO**	26.2 (29.9)	4.3 (11.1)	425047	24.5 (30.7)	1.5 (6.8)	482893
**16: AR, MS, LA**	29.1 (32.9)	3.3 (9.9)	685203	25.4 (31.3)	1.3 (7.1)	817341
**17: TX**	27 (29.6)	3.8 (8.7)	855235	26.4 (32.7)	1.6 (6.2)	750147
**19: MT, WY, UT, CO**	32.9 (41)	5.8 (15.5)	717531	27.3 (35.2)	2.1 (8.1)	590808
**20: WA, OR, ID, AK**	32.3 (37.4)	4.5 (16.7)	688514	26.1 (32.2)	3 (11.5)	502902
**21: CA, NV, HI, PH, GU, AS**	31.4 (36.1)	4.3 (11.5)	489036	25.4 (31.3)	1.9 (8.1)	684659
**22: CA, AZ, NM**	29.2 (29.3)	5.3 (11.7)	675112	28.4 (36.1)	2.2 (9.9)	934880
**23: ND, MN, SD, NE, IA**	25.7 (34.1)	2.9 (9.1)	628111	23.2 (30.2)	2.4 (11.9)	487169

Source: Veterans Health Administration's Corporate Data Warehouse. **Notes**: The table presents regression-adjusted estimates of median appointment wait times, controlling for regional differences in stop code mix. Standard deviations in parentheses. Primary states served are listed for each VISN. PH: Philippines; GU: Guam; AS: American Samoa.

© 2022 Feyman Y et al. *JAMA Network Open.*

eTable 6. Mean Appointment Wait Times and Referral Volumes, 2018-2019

Veterans Integrated Service Network (VISN)	Primary care: Mean wait (days) Community care	Primary care: Mean wait (days) VHA	Primary care: # of consults Community care	Primary care: # of consults VHA	Mental health: Mean wait (days) Community care	Mental health: Mean wait (days) VHA	Mental health: # of consults Community care	Mental health: # of consults VHA	All other specialties: Mean wait (days) Community care	All other specialties: Mean wait (days) VHA	All other specialties: # of consults Community care	All other specialties: # of consults VHA
**01: CT, MA, RI, NH, VT, ME**	46.3 (41.5)	24.2 (21.8)	1248	20566	33.9 (43.6)	27 (24.3)	1922	18388	33.7 (31.7)	30.6 (30)	129403	314638
**02: NY, NJ**	30.2 (28.9)	26.9 (22.3)	461	7134	37 (39.3)	31.5 (29)	976	12513	34.6 (28.8)	28.1 (25.2)	68502	331780
**04: PA, DE**	38.7 (28.9)	27.6 (26.1)	428	10566	35.5 (34.3)	27.8 (28.1)	1008	12006	33.5 (30.4)	31.6 (28.8)	145013	278015
**05: MD, DC, WV**	39.4 (39.6)	28.2 (26.2)	413	2240	63.4 (69.6)	39.2 (32.5)	2282	8688	42.6 (38.2)	32.7 (28.7)	87057	207528
**06: VA, NC**	48.2 (39.7)	24.9 (27.3)	8060	23236	56.4 (45)	39.7 (33.4)	4256	27498	51.9 (43.8)	37.5 (33.4)	246723	491816
**07: AL, GA, SC**	39.1 (31)	28.6 (29.5)	3186	9844	49 (44.8)	36.2 (30.7)	4950	23563	53.9 (47.6)	33.6 (30.3)	254038	440406
**08: FL, PR, VI**	35.7 (38.6)	39 (39.7)	461	20684	49.8 (52.8)	33 (29.5)	4853	32778	48.4 (43.2)	34.7 (32.9)	175350	701794
**09: KY, TN**	40.3 (32.2)	25.7 (21.4)	3704	4172	38.2 (34.2)	33.6 (28.4)	2866	16160	41.1 (31.2)	35.6 (29.9)	211242	293390
**10: OH, IN, MI**	39.4 (39.6)	23.3 (22.9)	3167	35870	42.3 (38.9)	30.9 (25.4)	3245	23232	38.3 (33.1)	29.3 (26.4)	209452	683232
**12: WI, IL**	37.1 (38)	20.9 (20.3)	1852	9997	48.5 (42.7)	23.5 (19.6)	1068	13724	36.9 (32)	30.6 (28.7)	145103	303228
**15: KS, MO**	30.1 (27)	41.2 (41.6)	2531	4251	36.1 (29.1)	32.2 (26.5)	3688	10049	30.9 (25)	30.1 (27.6)	186514	284612
**16: AR, MS, LA**	37.1 (37.5)	33 (32.7)	16372	7563	37.5 (36.3)	31.1 (26.5)	4325	19296	35.5 (30.9)	30.6 (28.3)	289855	513660
**17: TX**	27.1 (26.1)	28.9 (22.6)	13290	18884	41.5 (32.2)	29 (22.4)	6177	21492	33.7 (29.8)	30.8 (26.2)	389826	456751
**19: MT, WY, UT, CO**	30.1 (33.1)	27.4 (27.8)	3663	7679	47.5 (44.7)	30 (26.6)	6749	16026	41.1 (37)	33.7 (31.2)	305398	348481
**20: WA, OR, ID, AK**	31.2 (30.4)	27.9 (23.9)	22332	14390	37.5 (27.4)	26.4 (25.1)	9616	9228	36.1 (28.4)	31.5 (28)	310152	305199
**21: CA, NV, HI, PH, GU, AS**	33.6 (29.3)	23.9 (24.9)	1180	9149	40.3 (31.2)	30.7 (24.9)	5301	12624	37.4 (29.4)	30.5 (26.3)	208395	395799
**22: CA, AZ, NM**	33.1 (24.4)	30.8 (26.5)	7687	13665	37.1 (26.7)	39.3 (33.2)	17081	27663	34.9 (25)	34.4 (30.5)	302576	557414
**23: ND, MN, SD, NE, IA**	29.3 (35.4)	22.9 (23.2)	5282	6081	41.3 (35.2)	30.2 (27.7)	1953	13123	33 (30.4)	30.4 (31.1)	260823	280967

Source: Veterans Health Administration's (VHA) Corporate Data Warehouse. **Notes:** The table presents regression-adjusted estimates of mean appointment wait times, controlling for regional differences in stop code mix. Standard deviations in parentheses. Primary states served are listed for each VISN. PH: Philippines; GU: Guam; AS: American Samoa.
